# The carotid body: A novel key player in neuroimmune interactions

**DOI:** 10.3389/fimmu.2022.1033774

**Published:** 2022-10-24

**Authors:** Pedro L. Katayama, Isabela P. Leirão, Alexandre Kanashiro, José V. Menani, Daniel B. Zoccal, Débora S. A. Colombari, Eduardo Colombari

**Affiliations:** ^1^ Department of Physiology and Pathology, School of Dentistry, São Paulo State University, Araraquara, São Paulo, Brazil; ^2^ Department of Neurosciences and Behavior, Ribeirão Preto Medical School, University of São Paulo, Ribeirão Preto, São Paulo, Brazil

**Keywords:** Carotid body, inflammation, sympathetic nervous system, neuroimmunomodulation, neuroimmune interactions

## Abstract

The idea that the nervous system communicates with the immune system to regulate physiological and pathological processes is not new. However, there is still much to learn about how these interactions occur under different conditions. The carotid body (CB) is a sensory organ located in the neck, classically known as the primary sensor of the oxygen (O_2_) levels in the organism of mammals. When the partial pressure of O_2_ in the arterial blood falls, the CB alerts the brain which coordinates cardiorespiratory responses to ensure adequate O_2 _supply to all tissues and organs in the body. A growing body of evidence, however, has demonstrated that the CB is much more than an O_2_ sensor. Actually, the CB is a multimodal sensor with the extraordinary ability to detect a wide diversity of circulating molecules in the arterial blood, including inflammatory mediators. In this review, we introduce the literature supporting the role of the CB as a critical component of neuroimmune interactions. Based on ours and other studies, we propose a novel neuroimmune pathway in which the CB acts as a sensor of circulating inflammatory mediators and, in conditions of systemic inflammation, recruits a sympathetic-mediated counteracting mechanism that appears to be a protective response.

## Introduction

The nervous and immune systems interact to maintain physiological functions and to regulate pathological processes. These interactions occur in a bidirectional fashion – the nervous system can modulate the immune system and the immune cells and molecules can affect nervous system functions (1–7). Our understanding of neuroimmune interactions has substantially increased in the last years due to advancing technologies and combined efforts from the neuroscience, immunology, and physiology fields. It is currently known that local (i.e., within organs) and systemic (i.e., inter-organs) neuroimmune interactions govern many physiological functions as well as contribute to the development and maintenance of several diseases (8–11). Very illustrative examples of local neuroimmune interactions are those occurring within the gut, where its intrinsic nervous system (i.e., enteric nervous system) continuously modulates the activity of gut-resident immune cells to provide host defense against pathogens (12, 13). Differently, systemic neuroimmune interactions involve nervous and immune cells across different organs and can depend on central nervous system processing. For example, during acute systemic inflammation, the increased levels of circulating inflammatory mediators activate brainstem areas that are able to generate a counteracting protective response through changes in autonomic (i.e., parasympathetic and sympathetic) outflows that, in turn, modulate the function of immune cells within different peripheral organs to resolve inflammation (3, 7, 14–16).

In the latter example, because many immune cells and inflammatory molecules cannot readily cross the blood-brain barrier (17), some mechanisms have been identified to explain how brainstem areas could be activated in response to acute peripheral inflammation. In this context, the vagus nerve (X cranial nerve), a major component of the parasympathetic nervous system, was found to play an important role. The vagus is a mixed nerve, constituted by both afferent (from the body to the brain) and efferent (from the brain to the body) fibers, in a proportion of approximately 80% and 20%, respectively (18). Vagal afferent fibers carry important visceral sensory information from most peripheral organs to the central nervous system and, therefore, are essential players in the communication between the body and the brain (19). Regarding peripheral inflammation, several lines of evidence have indicated that vagal afferents are excited by circulating inflammatory mediators and relay this information to the central nervous system (1, 14, 20, 21). Indeed, it has been extensively reported that endogenous or exogenous stimulation of vagal afferents suppresses inflammation, likely by activating parasympathetic-related brainstem areas that generate a vagal efferent outflow to modulate peripheral immune cells (2, 7, 22–25).

Nevertheless, although the roles of vagal afferents as peripheral sensors of inflammation and vagal efferents as modulators of immune cells are well documented, much less is known about alternative, vagal-independent mechanisms in the context of systemic inflammation. An increasing body of new evidence has shown that several neuroimmune mechanisms do not depend on the vagus nerve or could compensate for its absence (3, 5, 16, 26). For instance, the intravenous administration of lipopolysaccharide (LPS) promoted systemic inflammation and increased splanchnic sympathetic nerve activity (3). This heightened sympathetic activity appears to be an anti-inflammatory response since in animals subjected to splanchnic sympathetic denervation, the LPS-induced inflammation was exacerbated, as reflected by higher levels of tumor necrosis factor-alpha (TNF-α) in the plasma (3). It is important to note that, although this effect was attributed to the lack of the inhibitory role played by splanchnic efferents on the splenic production of TNF-α (3), the absence of splanchnic afferents could have also contributed to exacerbating inflammation. Afferent fibers within the splanchnic nerves convey a great variety of sensory (mechanical, chemical, and noxious) information from visceral organs to the brain and, importantly, can be sensitized by inflammatory mediators (27, 28). Therefore, in the study of Martelli et al. (3), a contribution of splanchnic afferents to body-brain communication during LPS-induced systemic inflammation cannot be excluded. Another remarkable finding of the study was that bilateral vagotomy did not affect the LPS-induced increase in splanchnic sympathetic nerve activity nor the LPS-induced increase in TNF-α levels (3). Collectively, these findings support the existence of a neuroimmune mechanism that detects systemic inflammation and increases sympathetic outflow to modulate inflammation independently of vagal afferents (as peripheral sensors of inflammatory mediators) and vagal efferents (as neural modulators of peripheral immune cells). These results (3) also support a role of the sympathetic nervous system as a brain output in controlling inflammation (5, 16, 29, 30). However, a question remains: what could be the sensing mechanisms responsible for detecting systemic inflammation and driving the activation of brain circuits that increase sympathetic outflow to modulate the function of peripheral immune cells when the vagus nerves are absent?

Here, we review the literature supporting that the carotid bodies, a pair of small sensory organs located in the neck, are crucial players in body-brain communications in the context of inflammation. The carotid body (CB) is classically known as the primary sensor of the oxygen (O_2_) levels in the arterial blood, by sensing hypoxemia and activating brainstem autonomic areas to promote adequate cardiorespiratory adjustments to maintain homeostasis (31–33). However, it is now clear that the CB is much more than a hypoxemia/hypoxia sensor. Instead, the CB can monitor the levels of several molecules in the arterial blood (34, 35). This multisensorial ability confers to the CB the extraordinary capacity for integrating different systems, including interactions between the nervous and the immune systems. For instance, we recently found that the CB cells detect increased levels of TNF-α in the circulation and drives a sympathetic-mediated response to suppress systemic inflammation (26). Based on this and other studies, we propose that the CB is a key player in neuroimmune interactions and might be involved in the pathophysiology of inflammation-mediated diseases, representing a potential novel target for the development of new therapies.

## Carotid body anatomy and physiology

Before going into details about the role of the CB in neuroimmune interactions, a brief overview of CB anatomy and function is provided in this and the following section. The CB is a small paired organ located in the carotid bifurcations, the point where the common carotid artery divides into external and internal carotid arteries to supply blood to the brain (33, 34, 36, 37). This anatomical location is strategic: since the CB is the main sensor of the O_2_ levels in the body, one of its primordial tasks is monitoring the composition of the arterial blood flowing into the brain, which functioning is critically dependent on adequate O_2_ supply (35, 38). The CB is comprised by glomus cells (type I) and sustentacular cells (type II). The glomus cells are the most predominant and form clusters wrapped by sustentacular cells (33, 39). The glomus cells are responsible for detecting low levels of O_2_ in the arterial blood (hypoxemia), transducing this chemical information into neural signals that reach the central nervous system. In brief, when the partial pressure of O_2_ in the arterial blood falls, the CB glomus cells depolarize and release neurotransmitters that excite terminal afferents of the carotid sinus nerve (CSN) (34, 37, 38, 40). The CSN, which is a branch of the glossopharyngeal nerve (IX cranial nerve), conveys the signals from the CB to the nucleus tractus solitarius (NTS) in the brainstem (32, 37, 41). The NTS neurons, in turn, sends projections to brainstem areas related to the control of autonomic activity to promote cardiorespiratory adjustments to counteract hypoxemia and ensure the adequate delivery of O_2_ to the brain and body (32, 37, 42, 43).

The cardiorespiratory responses to hypoxemia include increases in arterial blood pressure and pulmonary ventilation and a decrease in heart rate (44, 45). The increase in arterial blood pressure results from a rapid elevation in sympathetic activity, driven mainly by a CB-mediated activation of NTS excitatory projections to the rostral ventrolateral medulla (RVLM), where most pre-sympathetic neurons are found (32, 46, 47). Notably, CB-mediated sympathetic effects during hypoxia are mostly excitatory, increasing lumbar, renal, and splanchnic sympathetic nerve activities (26, 48), only decreasing brown adipose tissue sympathetic nerve activity (49). Interestingly, CB stimulation by hypoxia also activates parasympathetic-controlling brainstem areas such as the nucleus ambiguus (NA) to increase parasympathetic activity resulting in bradycardia and, possibly, other effects (50–52). These observations demonstrate that the CB has a particular capacity for modulating both sympathetic and parasympathetic arms of autonomic outflows, impacting the function of different organs/systems. In the next section, we introduce the current concept that the CB contributes to regulating several body functions in a number of different conditions rather than only during hypoxia.

## Other functions of the carotid body: Much more than an O_2_ sensor

**Figure 1:**
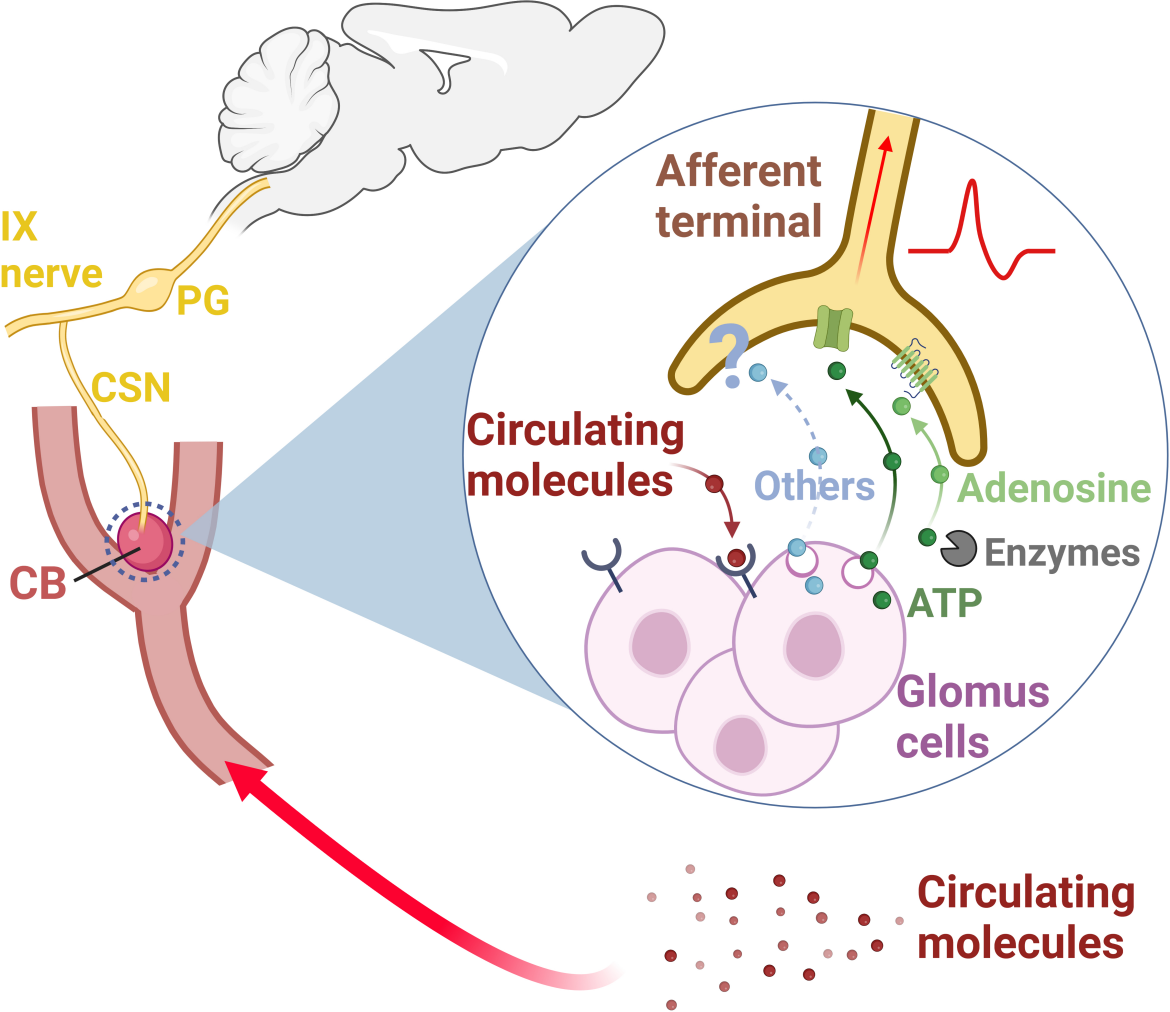
The carotid body (CB) is a sensory organ located at the carotid bifurcation (33, 34, 36). The carotid sinus nerve (CSN), a branch of the IX nerve (glossopharyngeal nerve), provides the sensory innervation to the CB (34, 36). Pseudo-unipolar neurons with cell bodies located within the petrosal ganglion (PG) convey the sensory information from the CB to the central nervous system (32, 34, 36, 37). This sensory information is multimodal, since the CB is responsive not only to changes in the partial pressure of oxygen in the arterial blood, but also to changes in the levels of several circulating molecules (epinephrine, angiotensin II, cytokines, endothelin, glucagon like peptide-1, glucose, insulin, leptin, and sodium chloride) (26, 53–55, 57–59, 61–64, 66, 67). Once depolarized, CB glomus cells release neurotransmitters, primarily ATP (39, 68). ATP binds to P2X2/P2X3 receptors in the afferent terminals of the CSN, generating action potentials which are propagated to the central nervous system (33, 38, 39, 68). Some of the ATP released by glomus cells is converted into adenosine by extracellular enzymes (NTPDase2,3 and ecto-5-nucleotidase) (38). Adenosine acts on A2a receptors on CSN afferent terminals, generating action potentials, and contributing to CB signaling to the central nervous system (38–40). Other neurotransmitters (Others) such as acetylcholine, dopamine, and serotonin, have been also found to modulate CSN activity (33, 37, 39). CB, carotid body; CSN, carotid sinus nerve; IX nerve, glossopharyngeal nerve; PG, petrosal ganglion. Created with BioRender.com.

Over the last 30 years, it has been increasingly discovered that the sensory function of the CB is not limited to hypoxia – besides those, the CB can detect an impressive variety of molecules (or their absence) in the circulation. For example, the CB is able to sense the plasma levels of angiotensin II (53), endothelin (54), leptin (55), cytokines (26, 56–59), epinephrine (60, 61), sodium chloride (62), glucose (63–65), insulin (66), and glucagon like peptide-1 (67) in the arterial blood. This very unique ability supports the current view that the CB is a multimodal sensory organ that monitors blood composition and informs the central nervous system on whether a given molecule is at low or high levels in the circulation (**Figure 1**). The central nervous system, in turn, organize the reflex adjustments, mainly through changes in autonomic outflows to regulate various body functions (34, 69).

One interesting example of a CB-mediated integrative homeostatic mechanism can be observed during hypoglycemia. Previous *in vitro* investigations have shown that CB glomus cells are sensitive to low levels of glucose (63, 64) encouraging later *in vivo* studies to explore the potential contribution of the CB in the homeostatic responses to hypoglycemia (61, 70). One of these studies, performed in humans, demonstrated that the hypoglycemia-induced acute release of counter-regulatory hormones that promote glycogenolysis and gluconeogenesis such as epinephrine, norepinephrine, cortisol, and glucagon is impaired by inhibition of the CB (70). The lower levels of epinephrine and norepinephrine in the plasma reflect a reduced sympathetic activation which, in turn, could have partially accounted for the lower levels of cortisol and glucagon when the CB is silenced (70). These findings illustrate how the CB plays an essential role in integrating different organs and systems. In this case, the CB acts as a sensor of blood glucose levels and coordinates a homeostatic response involving the sympathetic nervous and endocrine systems (70). In the following sections of this review, we focus on previous and new evidence indicating that the CB cells are also important for linking the nervous and the immune systems.

## Carotid body and neuroimmune interactions

In this section, we examine the growing body of evidence supporting that the CB is a sensor of peripheral inflammation and plays a pivotal role in recruiting a sympathetic-mediated anti-inflammatory response. As a starting point, it should be noted that the presence of receptors that allow for the recognition of pathogen-associated molecular patterns (PAMPs), damage-associated molecular patterns (DAMPs), and inflammatory mediators was reported in CB glomus cells and in PG neurons that provide CB sensory innervation. More precisely, it has been demonstrated that the mammalian CB and its innervating sensory neurons express toll-like receptors (TLR) 1 and 4, and receptors for inflammatory ligands such as TNF-α, interleukin 1-beta (IL-1β), interleukin 6 (IL-6), and lysophosphatidic acid (LPA) (26, 56, 71–75). Additional supportive evidence comes from studies showing that the CB is intrinsically responsive to several inflammatory mediators (56–58). For instance, whole-cell patch-clamp and calcium imaging experiments in cultured glomus cells demonstrated that IL-1β depolarizes the CB, inhibiting outward potassium currents and promoting calcium influx (58). In a recent study, it was shown that various pro-inflammatory cytokines (IL-4, IL-5, IL-13, IL-1β, IL-6, and TNF-α) and other inflammatory mediators (eotaxin and LPA) increase CSN activity in an isolated CB/CSN preparation (57). Notably, the same research group previously demonstrated that the LPA-induced CB activation stimulates phrenic and vagus nerve activities in an *in situ* decerebrated rat preparation (56), confirming that inflammatory mediators can directly activate the CB that, in turn, stimulates motor/autonomic outflows.

While these essential studies demonstrated that the CB has the machinery to intrinsically detect and respond to inflammatory mediators, other studies provided indirect but equally important evidence about the role of the CB in neuroimmune interactions, specifically in the context of systemic inflammation (71, 76, 77). In cats, the intravenous administration of LPS, resulting in systemic inflammation with high levels of various circulating pro-inflammatory cytokines, potently increased CSN discharge and caused tachypnea, tachycardia, and hypotension (71). Interestingly, the bilateral CSN section abolished the LPS-induced increase in respiratory frequency, indicating that the CB activates central respiratory pathways and stimulates breathing during systemic inflammation (71). A subsequent study provided further evidence that the CB is involved in brain-body communication during LPS-induced sepsis (76). The authors demonstrated that the intravenous administration of LPS in rats activates neurons in the NTS, the first relay site for CB afferents (76). Remarkably, LPS-induced NTS activation was profoundly suppressed in animals with bilateral CB denervation, strongly indicating that the CB is a crucial player in central nervous system activation during systemic inflammation (76). Furthermore, a later study from the same research group, suggested that the CB has a protective role during LPS-induced sepsis since bilateral CB denervation resulted in exacerbated plasma TNF-α, blunted plasma corticosterone, and faster progression to multiple organs dysfunction in comparison to rats with intact CBs (77).

Although the presented evidence seems sufficient to acknowledge the involvement of the CB in neuroimmune interactions, several questions still remain. Since intravenous administration of LPS increases the circulating levels of several inflammatory mediators such as TNF-α, IL1β, and IL-6 (78), what molecule (s) is (are) actually acting on the CB *in vivo*? What are the connections of the NTS neurons activated by CB afferents during inflammation? Is the CB-mediated activation of NTS neurons a counteracting response to systemic inflammation?

**Figure 2:**
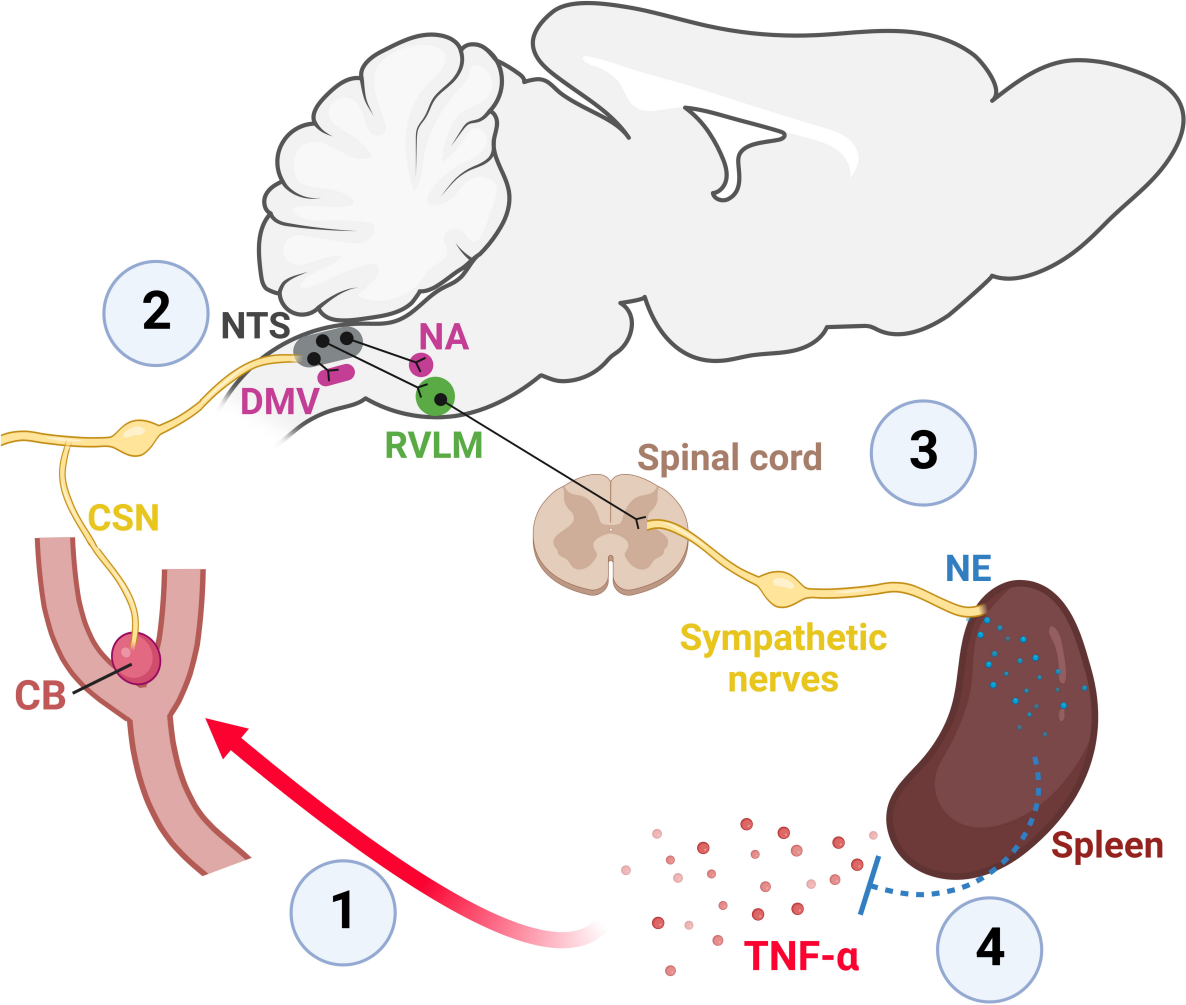
The CB is a sensor of peripheral inflammation and initiates a sympathetic-mediated anti-inflammatory response (26). 1: Elevated levels of TNF-α in the blood depolarizes CB glomus cells which widely express TNF-α receptors type I (26, 71–73). Depolarization of CB glomus cells by TNF-α generates action potentials that propagate along axons within the CSN towards the brainstem (26). 2: The first central synapse of CB-originated axons occurs in the NTS, a major integrative brainstem region that receives the sensory information from peripheral organs and projects to several brainstem autonomic areas that control parasympathetic (DMV and NA) and sympathetic (RVLM) functions (32, 41, 46, 50, 52). 3: The TNF-α-induced activation of the CB-NTS-RVLM circuit increases the activity of the splanchnic sympathetic nerve which innervates the celiac ganglia, from where the splenic nerve originates and projects to the spleen, releasing norepinephrine (NE) (26). 4: The release of NE into the spleen reduces both splenic and plasmatic levels of TNF-α (26). CB, carotid body; CSN, carotid sinus nerve; DMV, the dorsal motor nucleus of the vagus; NA, nucleus ambiguus; NTS, nucleus tractus solitarius; NE, norepinephrine; RVLM, rostral ventrolateral medulla; TNF-α, tumor necrosis factor-alpha. Created with BioRender.com.

Therefore, in a recent study, we sought to further dissect the role of the CB in neuroimmune interactions (26). More specifically, we investigated if the CB could detect increased levels of TNF-α in the circulation and activate central autonomic pathways to potentially modulate inflammation. We decided to focus on TNF-α because its type I receptor (TNFR1) was already reported to be found in CB glomus cells of several mammalian species (including rats) (71, 73, 79) and, also, because TNF-α is a well-known critical mediator of inflammation (80). We first examined and confirmed that the TNF-α receptor type I is expressed in the CB glomus cells of rats at mRNA and protein levels (26). Next, we found that the intravenous administration of TNF-α increased CSN afferent discharge (26), suggesting that the CB can detect increased levels of TNF-α in the circulation. Since the first central synapse of CB afferents occurs in the NTS (32, 81), and because CB stimulation by hypoxia activates monosynaptic excitatory projections from NTS to RVLM, increasing sympathetic outflow (32, 46), we hypothesized that the observed TNF-α-induced increase in CSN afferent activity could stimulate this pathway (26). We found that intravenous TNF-α activated a high number of RVLM-projecting NTS glutamatergic neurons and that this activation was dependent on CB input since the number of activated neurons was drastically reduced in rats previously subjected to bilateral CB ablation (26). These results suggest that the CB detects circulating TNF-α and activates a central sympathoexcitatory pathway. Consistent with the observation that circulating TNF-α activates the CB to recruit a NTS-RVLM sympathoexcitatory pathway, we found that systemic TNF-α increased splanchnic sympathetic nerve activity, and importantly, CB ablation strongly attenuated this response (26). This observation further supports the role of the CB as a sensor of circulating TNF-α and a key player in body-brain communication in this model. Finally, because the splanchnic sympathetic nerve was shown to exert an anti-inflammatory and protective role during systemic inflammation (3, 15, 16), we hypothesized that the TNF-α-induced activation of the sympathetic circuits and splanchnic sympathetic nerve activity would counteract inflammation. Confirming this assumption, the levels of cytokines measured in the plasma and in the spleen 2 hours after the TNF-α administration were significantly increased in animals previously subjected to CB removal or splanchnic sympathetic denervation compared to sham animals (26). In summary, our results suggest a novel neuroimmune mechanism in which the CB acts as a sensor of peripheral inflammation, detecting increased levels of circulating TNF-α and communicating with the brain to activate a counteracting anti-inflammatory reflex mediated by the splanchnic sympathetic nerve. A schematic illustration of the novel mechanism is presented in **Figure 2**.

The discovery of this novel neuroimmune mechanism raises numerous possibilities and questions. As well pointed out by a recent report (82): could other sympathetic nerves (besides the splanchnic) and/or premotor sympathetic areas (besides the RVLM) also contribute to regulating inflammation? In addition, we also add the following questions: What is the role of this mechanism in chronic inflammatory conditions? Are CB-mediated activation of NTS projections to brainstem parasympathetic nuclei (DMV and NA) activated during inflammation? Does the parasympathetic nervous system also play a role in this neuroimmune mechanism? Does chronic inflammation cause CB-mediated sympathetic overactivation in disease states? Although further investigations are needed to answer all the raised questions described above, in the next section of this review, we briefly discuss the potential role of inflammation in the pathological overactivation of the CB observed in several diseases.

## Targeting the CB as a therapeutic approach for inflammatory diseases?

Because of its multisensory ability and integrative role, it is somewhat expected that a CB dysfunction could impact the activity of different organs/systems and, thus, contribute to various diseases. Numerous pre-clinical and clinical studies have shown that CB dysfunction, usually characterized by its overactivity, plays a role in hypertension (83–85), heart failure (86, 87), apneas of prematurity (88, 89), and metabolic diseases (90, 91). In hypertension and heart failure, for example, a chronic CB overactivation has been associated with the sustained activation of the sympathetic nervous system, which contributes to the development and maintenance of these conditions (83, 87, 92–95).

Over the last decade, the surgical removal of the CB has been considered a therapeutic option to overcome this CB-mediated sympathetic overactivation in hypertension (83, 85, 92, 96) and heart failure (86, 95). Although the outcomes of these studies were very promising, concerns about the safety of removing the CBs were raised (45, 97). In spontaneously hypertensive rats, the bilateral CB removal abolished the pressor responses to hypoxia and exacerbated the pressor and respiratory responses to hypercapnia, indicating an impaired capacity for regulating cardiorespiratory functions in CB-ablated animals (45). In addition, based on the supposed protective role of the CB during systemic inflammation and sepsis (26, 77), CB ablation could result in catastrophic effects if the individual is exposed to these conditions.

In this context, pharmacological approaches have been described (93, 98) as promising options for reducing CB overactivity. However, to the best of our knowledge, there are no reports considering the targeting of inflammatory signaling within the CB in diseases such as hypertension and heart failure. Since a chronic state of inflammation is a common feature in these conditions (99, 100) and because inflammation-related molecules activate the CB (26, 57), we hypothesize that chronic inflammation contributes to the excessive CB activation observed in some diseases and thus, propose that targeting inflammation within the CB might represent a new therapeutic approach. However, studies are needed to test this hypothesis.

If, on one side, a great effort has been made to suppress CB activity in chronic conditions such as hypertension and heart failure, on the other side, much less is known about strategies to enhance CB activity that could be favorable in some situations such as in acute systemic inflammation (26, 77, 101, 102). Preclinical studies conducted in rodents have provided exciting evidence for considering the electrical stimulation of the CB to counteract acute systemic inflammation (101, 102). One of these studies employed a technique that allows the electrical stimulation of the carotid sinus/CSN in unanesthetized rats (103) and found that CB activation attenuates LPS-induced systemic inflammation likely by recruiting parasympathetic- and sympathetic-mediated mechanisms (101). Another study demonstrated that CSN stimulation in mice efficiently reduced LPS-induced inflammation by activating the hypothalamus-pituitary-adrenal axis, promoting corticosterone release, which, in turn, suppressed the activity of myeloid cells (102). Nevertheless, some questions remain. First, which type(s) of fiber(s) in the CSN (A-fibers, C-fibers, or both) mediate the observed anti-inflammatory effects following CB/CSN electrical stimulation in the abovementioned studies? Second, could different electrical stimulation parameters selectively activate CSN A-fibers, C-fibers, or both? Last, would the activation of specific CSN fiber types promote differential effects on the control of inflammation? For example, studies conducted on electrical stimulation of the vagus nerve have demonstrated that specific stimulation parameters can recruit different fiber types (104, 105). Moreover, it has been shown that specific parameters for electrical stimulation of the vagus nerve results in differential effects on circulating cytokines (24). Therefore, although the outcomes of CB activation in rodents to modulate inflammation seem encouraging, the translation of electrical stimulation of the CB/CSN to the clinical scenario still requires further investigation.

## Challenges of CB stimulation as a therapeutic strategy

Besides the need for more robust evidence on the mechanisms underlying the electrical activation of the CB, as discussed above, safety concerns are probably the main challenge for implementing this approach in clinical settings. A surgical, invasive approach is necessary to access the CB and the CSN. Since the CB and surrounding areas are highly vascularized and innervated (34, 106), potential inaccuracies during the surgical procedure could result in serious consequences. In this context, ultrasound-based neural stimulation could be explored as a non-invasive alternative for activating the CB/CSN. Recent studies have shown successful applications of ultrasound stimulation for targeting neural pathways within specific organs, such as the spleen and the liver, modulating inflammation and glucose homeostasis, respectively (107, 108). A challenge for this, however, may rely on the fact that the carotid baroreceptor fibers and CB fibers are in close proximity and run together within the CSN (34, 109), making it very difficult to specifically target one and not the other.

## Conclusion

In this review, we highlight the CB as a unique organ with extraordinary capacities for sensing a great variety of molecules and integrating different organs/systems. We focus on the role of the CB in mediating the integration between the nervous and the immune systems. In this context, we introduce a novel mechanism of neuroimmune interaction in which the CB acts as a sensor of inflammatory ligands in the circulation and recruits central sympathetic networks that counteract inflammation. Furthermore, based on a growing body of preclinical and clinical research, we propose new perspectives on managing inflammatory diseases by targeting the CB.

It should be noted that although most of the studies discussed in this review reported a sympathetic-mediated suppression of immune responses (3, 5, 16, 26), the sympathetic nervous system, in some conditions, can actually activate the immune system (110–112). Therefore, despite the potential therapeutic possibilities highlighted in the present study seem promising, there is still a lot to explore regarding the intricate interactions between the nervous and immune systems.

## Author contributions

PK conceived and designed the manuscript. PK, IL, AK, JM, DZ, DC, and EC drafted and revised the manuscript. PK prepared the figures. All authors read and approved the final version of the manuscript.

## Acknowledgments

This work was supported by the Sao Paulo Research Foundation (FAPESP; grants #2019/11196-0, #2015/23467-7, and #2019/24154-3) and PROPG-UNESP. Figures were created with BioRender.com.

## Conflict of interest

The authors declare that the research was conducted in the absence of any commercial or financial relationships that could be construed as a potential conflict of interest.

## Publisher’s note

All claims expressed in this article are solely those of the authors and do not necessarily represent those of their affiliated organizations, or those of the publisher, the editors and the reviewers. Any product that may be evaluated in this article, or claim that may be made by its manufacturer, is not guaranteed or endorsed by the publisher.
